# Protecting healthcare workers and patients during the COVID-19 pandemic: a comparison of baseline and follow-up infection prevention and control needs in Nigerian military healthcare facilities delivering HIV services

**DOI:** 10.1186/s12913-023-10289-x

**Published:** 2023-11-14

**Authors:** Elizabeth H. Lee, Ayesha Rashid, Ismail Lawal, Usman Adekanye, Yakubu Adamu, Catherine Godfrey, Patricia A. Agaba, Nathan Okeji, Priyanka Desai

**Affiliations:** 1 Department of Pediatrics, Uniformed Services University of the Health Sciences, Bethesda, MD USA; 2 US Military HIV Research Program, Walter Reed Army Institute of Research, Silver Spring, MD USA; 3 US Army Medical Research Directorate – Africa/Nigeria, Walter Reed Army Institute of Research, Abuja, Nigeria; 4 Nigerian Ministry of Defence - Health Implementation Programme, Abuja, Nigeria; 5 Office of the Global AIDS Coordinator, Department of State, US President’s Emergency Plan for AIDS Relief, Washington, DC USA; 6 Henry M. Jackson Foundation for the Advancement of Military Medicine, Bethesda, MD USA

**Keywords:** Infection prevention and control, COVID-19, Pandemic preparedness, Needs assessment

## Abstract

**Background:**

Protecting the HIV health workforce is critical for continuity of services for people living with HIV, particularly during a pandemic. Early in the COVID-19 pandemic, the Nigerian Ministry of Defence, in partnership with the US Military HIV Research Program, took steps to improve infection prevention and control (IPC) practices among staff working in select PEPFAR-supported Nigerian military health facilities.

**Methods:**

We identified a set of IPC activities a priori for implementation at four Nigerian military hospitals in HIV and related departments in early 2021, including continuous medical masking, physical distancing, placement of additional hand washing stations and hand sanitizers throughout facilities, and training. We fine-tuned planned intervention activities through a baseline needs assessment conducted in December 2020 that covered eight IPC components: ‘IPC program structure, funding and leadership engagement’; ‘IPC policies, guidelines and standard operating procedures (SOPs)’; ‘infrastructure’; ‘triage and screening’; ‘training, knowledge and practice’; ‘personal protective equipment (PPE) materials, availability and adequacy’; ‘biosafety and waste management’; and ‘monitoring and remediation’ prior to implementation. Baseline results were compared with those of a follow up assessment administered in August 2021, following intervention implementation.

**Results:**

IPC readiness remained high at both baseline and follow-up assessments for ‘IPC guidelines, policies, and SOPs’ (96.7%). The components ‘infrastructure’ and ‘monitoring and remediation’, which needed improvement at baseline, saw modest improvements at follow-up, by 2% and 7.5%, respectively. At follow-up, declines from high scoring at baseline were seen in ‘IPC program structure, funding and leadership engagement’, ‘training, knowledge and practice’, and ‘biosafety and waste management’. ‘PPE materials availability and adequacy’ improved to 88.9% at follow-up. Although unidirectional client flow was newly implemented, the score for ‘triage and screening’ did not change from baseline to follow-up (73%).

**Conclusion:**

Variability in IPC component readiness and across facilities highlights the importance of building resilience and employing a quality improvement approach to IPC that includes regular monitoring, re-assessment and re-training at set intervals. Results can be used to encourage solutions-oriented dialogue between staff and leadership, determine needs and implement action plans to protect staff and people with HIV.

**Supplementary Information:**

The online version contains supplementary material available at 10.1186/s12913-023-10289-x.

## Introduction

Recent pandemics like coronavirus disease 2019 (COVID-19) and outbreaks such as Ebola Virus Disease (EVD) and mpox highlight the continued importance of public health measures and health systems preparedness to be responsive to infectious disease outbreaks. The global impact of COVID-19 has demonstrated the need to protect front-line healthcare workers (HCWs) who are at high risk of infection exposure in the healthcare setting through evidence-based infection prevention and control (IPC) measures. Effective IPC at every patient-provider interaction is fundamental to ensuring safe, quality healthcare services, and requires unwavering commitment by all stakeholders at all health system levels [1]. The World Health Organization (WHO) defines a set of core components for successful IPC as: programs; guidelines; education and training; healthcare-associated infection (HCAI) surveillance; multimodal strategies; monitoring, audit and feedback; workload, staffing and bed occupancy at the facility level; and built environment, materials and equipment at the facility level [2].

HCWs were the backbone of the COVID-19 response early on when emergency rooms were overwhelmed with severe illness. Protecting HCWs worldwide quickly became a priority, and particularly for low- and middle-income countries the predictions about the secondary impacts on medical hygiene and service delivery pointed to the fragility of the health systems. In June of 2020, the WHO reported a total of 5,766 health worker cases across 37 countries that accounted for 26% of all reported cases [3]. National surveillance in Nigeria suggested that HCWs made up an alarming 9.3% of confirmed COVID-19 cases (n = 1,139) in the first six months of the pandemic, and early assessment of Nigerian facilities showed low IPC preparedness [4, 5].

Across the African continent, human immunodeficiency virus (HIV) prevention and treatment services were deeply impacted by the COVID-19 pandemic [6]. The U.S. President’s Emergency Plan for AIDS Relief (PEPFAR), a US government commitment to ending the HIV pandemic, recognized the need to protect health systems in partner countries while maintaining focus on the core mandate. PEPFAR rapidly disseminated guidelines to protect HCWs and immuno-compromised people living with HIV (PLHIV) early in the COVID-19 pandemic in concert with national policies and guidelines across PEPFAR-supported countries to ensure uninterrupted HIV service delivery [7]. Putting this guidance into practice was further made possible by PEPFAR’s approval to (1) pause HIV prevention campaign activities and new HIV surveillance activity scale-up, (2) use differentiated models of care, including expanded access to multi-month ART dispensing with the aim of decongesting facilities to protect PLHIV and HCWs from exposure where national policies allowed, and (3) provide support for evidence-based measures designed to reduce exposures in the workplace [8].

As part of early efforts to protect gains in PEPFAR-supported health systems, we designed and implemented a proof-of-concept IPC intervention a year into the pandemic using a quality improvement lens at a set of healthcare facilities in collaboration with the Nigerian military. Our short-term goal was to improve IPC for SARS-CoV-2 and other respiratory infections among staff working in select PEPFAR-supported Nigerian military health facilities, with a longer-term goal of rolling out the intervention to other PEPFAR-supported national HIV programs. We describe results of baseline and follow-up assessments before and nearly seven months after intervention initiation to understand changes in facility IPC readiness in the second year of the COVID-19 pandemic.

## Methods

### Context

The U.S. Military HIV Research Program (MHRP) at Walter Reed Army Institute of Research provides technical assistance to the Nigerian Ministry of Defence Health Implementation Programme (NMOD-HIP) in HIV prevention, care and treatment and related co-morbidities at forty military-operated health facilities serving over 36,000 PLHIV or people at risk for HIV in Nigeria. Health facilities provide inpatient and outpatient services across the Army, Navy, and Air Force tri-services. MHRP also supports over 180 personnel in these facilities, representing a significant investment in human resources for health. Facilities are located on military bases and provide services to active-duty service members, dependents, and civilians from the surrounding catchment area. The size and scope of the HIV program, previous experience in EVD and Lassa outbreaks, as well as the command and control structure of the military, made intervention in this setting feasible in a short time frame [7, 9].

### Facility selection

Facilities were eligible for intervention and assessment if they were tertiary hospitals with > 300 clients active on ART, located in a state with high COVID-19 burden reported by the Nigeria Centre for Disease Control and Prevention as of September 2020, had a COVID-19 isolation center, and were in close proximity to a laboratory capable of testing for COVID-19 by polymerase chain reaction. Facility research experience and inclusion of at least one facility from each of the tri-services were also considerations.

### Intervention design and implementation

**Fig. 1:**
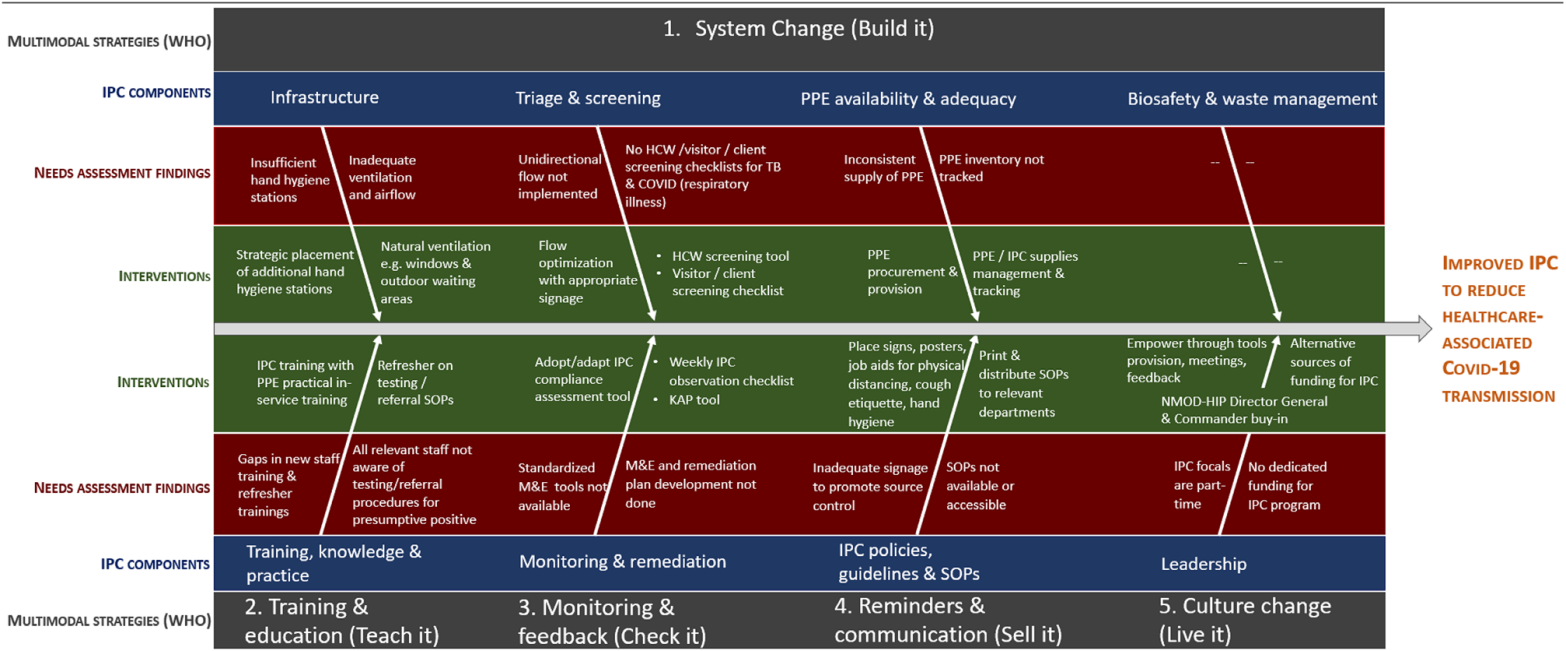
Fishbone diagram of IPC gaps mapped to intervention elements. Fig. 1 depicts the overall framework for the IPC intervention with gaps mapped to multimodal strategies, IPC components, and each intervention element

Based on evolving evidence at the start of the COVID-19 pandemic, we identified a set of IPC activities *a priori* based on knowledge of the service delivery environment, CQI principles, examples from the gray and peer-reviewed literature, and modified WHO IPC components to design a multimodal, structured intervention. Eight, integrated IPC intervention components selected for the implementation environment were: ‘IPC program structure, funding and leadership engagement’; ‘IPC policies, guidelines and standard operating procedures (SOPs)’; ‘infrastructure’; ‘triage and screening’; ‘training, knowledge and practice’; ‘PPE materials, availability and adequacy’; ‘biosafety and waste management’; and ‘monitoring and remediation’ (Fig. 1). We then confirmed need for and fine-tuned intervention elements through a baseline needs assessment in December 2020.

Priority needs identified for intervention included personal protective behaviors (PPBs) such as continuous medical masking, face coverings for all, hand hygiene and physical distancing. Systems-level changes incorporated into the intervention to enable these PPBs were multifaceted. To increase the practice of and accessibility to hand-hygiene, additional hand washing stations and hand sanitizers were placed at appropriate locations throughout facilities such as entry and exit points, patient waiting areas and points of care. Client flow was optimized by posting signs and directions to supplement NMOD-HIP efforts such as tents to expand waiting areas and other engineering and administrative controls. In addition, service flow was aligned within the existing facility footprint so that from the facility entrance patients were triaged to the appropriate service unit (e.g. emergency, outpatient, etc.), followed by pharmacy and records units for prescription pick up and follow up appointment booking where necessary; no services were physically relocated. We modified a US CDC COVID-19 questionnaire so that all staff could self-screen daily for exposures and symptoms, and to prompt testing and isolation and quarantine [10]. Testing frequency, results and referral for isolation, quarantine and treatment data were not collected. Consistent availability and tracking of supplies and PPE via an inventory tracking tool that captured distribution of commodities was also instituted. Medical masks including N95 respirators were made sufficiently available and distributed from a central point within the facility to promote continuous medical masking by all staff. Additional PPE equipment such as face shields, goggles, gloves and gowns were provided to the facilities to further enhance IPC measures for those coming in close contact with and handling of potential sources of infection. Additionally, in-service training and planned routine refreshers were integral elements of the intervention, with emphasis on augmenting clinical and laboratory staff knowledge, although all intervention participants were trained.

The proof-of-concept IPC intervention was led by IPC focal persons and implemented across HIV, general outpatient, accident and emergency and administrative service areas from mid-January 2021 through mid-April 2021. Staff from across laboratory, environmental and occupational health, and janitorial services participated, given our holistic, systems-oriented approach to reducing airborne disease transmission and desire to permanently embed the intervention at facilities and create a protective cultural shift. In line with a quality improvement approach and the IPC component ‘monitoring and remediation’, we designed several tools to monitor intervention implementation over time to inform remediation as appropriate. These included the needs assessment tool which was repeated at specified intervals. Other tools included a weekly observation checklist to monitor compliance with IPC procedures and guidelines, as well as a knowledge, attitudes and practices survey administered to the participating staff (e.g. clinical, administrative, janitorial). Here, we describe and present findings from the needs assessment at baseline and first follow-up after intervention initiation.

### Needs assessment questionnaire design, data collection and analysis

The needs assessment questionnaire was based upon questions adapted from publicly-available tools from the gray literature that were relevant for the Nigerian military in the context of COVID-19 [11–16]. Questions asked about then-current COVID-19 epidemiology, national policy dissemination, as well as the eight IPC components using a yes/no structure, and additional space was provided for open-ended written reflections. Baseline assessments using the same questionnaire were conducted at participating sites in December 2020 and followed up again in August 2021 using the same tool to document IPC practices and ongoing needs.

A single individual administered the paper questionnaire to preexisting IPC focal persons at each facility. Each question was worth a total of one point for a response of yes or zero points for response of no. The eight IPC components were scored as a simple average of the scored questions within, and results were calculated as a percent score for each component by facility, as well as overall for all facilities. Components and questions were color coded to facilitate staff use of data for quality improvement (Additional file 1 and 2). We calculated the absolute percent difference for baseline and follow-up component scores. Results were blinded to protect military site confidentiality.

### Costing analysis

We also carried out a costing analysis over the life of the project that included the intervention implementation, and monitoring and evaluation activities including needs assessment. We estimated average total cost, cost per facility and unit cost per staff member for each facility during the evaluation period. Costs included estimated level of effort for HCWs and support staff based on self-report, and by average cadre salary. US Department of Defense- supported program staff who provided technical assistance to sites provided time in-kind. Other costs were those incurred during in-service training, travel for supportive supervision visits and procuring IPC supplies. The cost of certain PPE supplies were included (surgical masks, N95 respirators, face shields, googles, gloves, gowns) as well as sanitizers, disinfectants, thermometers and laboratory and clinical supplies.

## Results

10% (4 out of 40) of NMOD-HIP facilities from three states met selection criteria and agreed to participate. Two facilities were located in Lagos state, a third in the Federal Capital Territory (FCT) and the fourth in Kaduna state. In January 2021, these states accounted for more than half of all reported COVID-19 cases in Nigeria [17]. The facilities represented all three uniformed services branches under the Nigerian Armed Forces: the Army, Navy and Air Force. The facilities had previously participated in disease-specific training, as well as trainings on biosecurity, biosafety, procurement and use of PPE, pathogen containment, pathogen storage, and prevention of pathogen spread. Facilities each had an existing IPC committee and focal person responsible for ensuring adherence to IPC policies and practices. Facilities were also part of a robust HIV continuous quality improvement (CQI) and quality management culture.

**Fig. 2:**
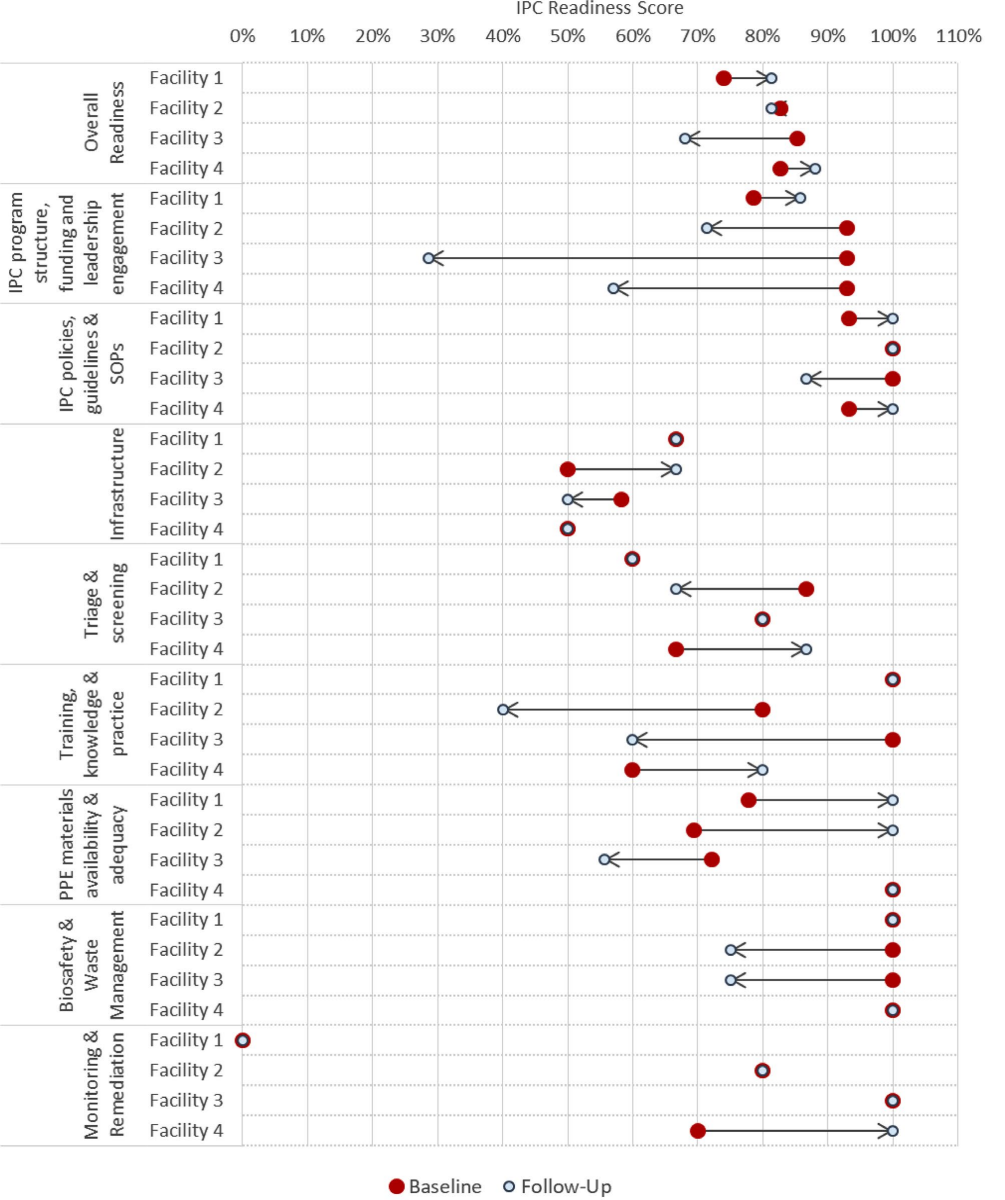
Baseline and Follow-Up IPC Readiness Scores: By IPC Component Overall and for Each Facility. Fig. 2 shows the scores for infection prevention and control (IPC) readiness overall and by IPC component, for each facility, at baseline and follow-up assessment, as well as the direction of change

Across all components, average IPC readiness declined slightly from 81.2% at baseline to 79.7% at the follow-up assessment (Table 1). IPC readiness was variable by time point of assessment, IPC component, facility, and certain individual questions that rolled up into aggregate component scores. Facility 4 was the only facility to increase scores across four of the eight IPC components (Fig. 2). By contrast, Facility 3 had a drop in performance for six of the eight IPC components at follow-up compared to baseline. Below, we present a comparison of baseline and follow-up assessment results by IPC component and in relation to individual question responses.

**Table 1:** Baseline and follow-up assessment scores: IPC readiness overall and by component

	Baseline Value, %	Follow-Up Value, %	Difference, %
Overall Readiness	81.2%	79.7%	-1.5%
IPC program structure, funding and leadership engagement	89.3%	60.7%	-28.6%
IPC policies, guidelines and SOPs	96.7%	96.7%	--
Infrastructure	56.3%	58.3%	2.0%
Triage and screening	73.3%	73.3%	--
Training, knowledge and practice	85.0%	70.0%	-15.0%
PPE materials availability and adequacy	79.9%	88.9%	9.0%
Biosafety and waste management	100.0%	87.5%	-12.5%
Monitoring and remediation	62.5%	70.0%	7.5%

Table 1 provides average infection prevention and control (IPC) readiness scores at baseline and follow-up assessment (bolded) as well as the absolute percent difference for participating facilities overall (n = 4), as well as for each of the eight IPC components

### IPC program structure, funding and leadership engagement

Overall, ‘IPC program structure, funding, and leadership engagement’ for facilities declined from a baseline score of 89.3–60.7% on follow-up. Although one facility improved, the others declined, and this decline was substantial for Facility 3 (Additional file 2). All facilities reported having an IPC program and designated focal person at baseline, but during follow-up Facility 3 indicated this was no longer the case. Lack of a dedicated budget for IPC program support was cited as challenging in open response text. Facilities 2 and 3 also indicated routine engagement of facility leadership with the IPC committee was lacking at follow-up.

### IPC policies, guidelines and SOPs

‘IPC Policies Guidelines and SOPs’ consistently maintained a high readiness score of 96.7% at baseline and follow-up, with COVID-19 guidelines and SOPs on topics such as PPE use, hand hygiene remaining available across all facilities. Two facilities improved to perfect availability, while one declined to 86.7% due to difficulties in maintaining physical distancing guidance among clients and easily accessible COVID-19 SOPs.

### Infrastructure

The ‘infrastructure’ component of IPC readiness had the lowest score at both baseline and follow-up, with an average score of 56.3% and 58.3%, respectively. While there were some improvements on individual questions and overall for Facility 2, Facilities 1 and 4 saw no change, and Facility 3 declined. All facilities had functional hand hygiene stations at entry, exit and points of care, but adequacy of ventilation varied at baseline. At follow-up, all facilities reported adequate ventilation due in part to pitching of tents outdoors to increase waiting area capacity, but none had air filtration or UV irradiation systems outside of COVID-19 treatment areas.

### Triage and screening

Scores for ‘triage and screening’ remained at 73.3% from baseline to follow-up, with two facilities showing no change, one improving and one decreasing. The varied performance on questions regarding unidirectional flow, symptom screening practices, tools and documentation for facility staff and visitors drove overall scoring. All facilities reported screening visitors and patients for COVID-19 consistently from baseline to follow-up, but no facilities reported documenting HCW screening responses at either time point despite doing so during the intervention period.

### Training, knowledge and practice

The ‘training, knowledge, and practice’ component of IPC readiness declined from 85% at baseline to 70% at follow-up. Facility 1 maintained a perfect score, Facility 4 improved substantially, while Facilities 2 and 3 declined significantly. All facilities reported providing training on COVID-19 IPC, hand-hygiene practices, and standard precautions, but new staff orientation and refresher training were challenging areas. Training records were available at all sites at baseline but only at Facility 1 at follow-up. Facility 4 had improved its inclusion of IPC training in new staff orientation and had conducted IPC refresher training since intervention initiation. Facilities 2 and 4 reported deficits on these training items at follow-up.

### PPE materials availability and adequacy

To score ‘PPE materials availability and adequacy’, we asked what PPE and IPC items were available at assessment (gloves, medical masks, face shields or goggles, soap or alcohol rub, surface disinfectant, single use towels), and whether there were challenges with requesting each. We also asked about whether inventorying, logging when dispensed, reordering using minimum levels, and forecasting was conducted. Scoring of ‘PPE materials availability and adequacy’ improved from a baseline of 79.9–88.9% at follow-up, with three facilities scoring perfectly at follow-up. Most facilities reported immediate availability of a range of PPE and cleaning supplies with no major challenges accessing these materials. However, Facility 3 declined substantially to 55.6%, reporting no availability of eye protection or paper towels, as well as challenges with mask and glove supply. All facilities maintained an inventory and tracked commodities in a log book; however, at follow-up, Facility 3 still had not established minimum reorder levels nor conducted forecasting.

### Biosafety and waste management

Overall, ‘Biosafety and waste management’ readiness was high, with an average score of 100% at baseline that declined to 87.5% at follow-up. Facilities 1 and 4 maintained perfect scores, while Facility 2 and 3 dropped from 100 to 75% at follow-up. All facilities had documented waste management procedures including materials for waste segregation and access to waste treatment at baseline and follow-up. All but Facility 2 reported maintaining training across all staff (including cleaning staff) for proper waste segregation and disposal at baseline and follow-up; Facility 2 reported a change in this status at follow-up assessment. All facilities had adequate waste bins or other containers to segregate waste at baseline; only Facility 3 reported inadequate bins/containers at follow-up.

### Monitoring and remediation

On average, ‘monitoring and remediation’ readiness increased slightly from 62.5% at baseline to 70% at follow-up. Facility 3 scored 100%, while Facility 2 scored 80% with no use of standardized tools/checklists. Facility 4 showed improvement from 70 to 100% at follow-up, with successful implementation of standardized monitoring and evaluation tools and reporting and remediation plan efforts. Facility 1 reported no implementation across all questions at baseline and follow-up.

### Costing results

The total cost of the intervention and evaluation for all facilities was ₦8,432,721 or $20,238.53 using an exchange rate of 1 Nigerian Naira = 0.0024 USD over three months (Table 2). Costs per facility ranged from a low of $2,482.62 at Facility 2 to a high of $7,865.67 at Facility 1. Cost per participant was an average of $79.68 over three months, ranging from a low of $43.95 at Facility 3 to a high of $131.85 at Facility 4. The average cost per participant for one month was $26.56, and projected out for 12 months was $318.72.

**Table 2:** Calculation of total costs by type (in Nigerian Naira (NGN)* and US dollar (USD)

	**Costs and proportion [n (%)] of total by Facility and Overall** **in Nigerian Naira (NGN), unless US Dollars (USD) indicated**					
**Cost type**	**Facility 1**	**Facility 2**	**Facility 3**	**Facility 4**	**Total**	
Facility Staff	560,500 (26)	529,600 (25)	549,250 (26)	500,750 (23)	2,140,100 (100)	
Training	798,100 (34)	104,000 (4)	506,500 (22)	917,800 (39)	2,326,400 (100)	
Supportive Supervision	685,113 (45)	30,000 (2)	183,690 (12)	623,268 (41)	1,522,071 (100)	
Supplies and Equipment	1,213,649 (51)	350,825 (15)	269,477 (11)	525,199 (22)	2,359,150 (100)	
Shared Resources	20,000 (24)	20,000 (24)	30,000 (35)	15,000 (18)	85,000 (100)	
Total NGN	3,277,362 (39)	1,034,425 (12)	1,538,917 (18)	2,582,017 (31)	8,432,721 (100)	
**Total (USD)**	**7865.67 (39)**	**2482.62 (12)**	**3693.40 (18)**	**6196.84 (31)**	**20238.53 (100)**	
Enrolled participants (count)	72	51	84	47	254	
Cost/ participant	45518.92	20282.84	18320.44	54936.53	33199.69	
**Cost/participant (** ***USD*** **)**	**109.25**	**48.68**	**43.97**	**131.85**	**79.68**	
Cost/month/participant (*USD*)	36.42	16.23	14.66	43.95	26.56	
Cost/year/participant (USD)	436.98	194.72	175.88	527.39	318.72	

Table 2 depicts the total cost of the intervention overall and by facility (bolded), as well as broken out by cost type, in Nigerian Naira and United States Dollars. The total cost per participant in USD is also bolded. *1 Nigerian Naira = 0.0024 US average in 2022

**Fig. 3:**
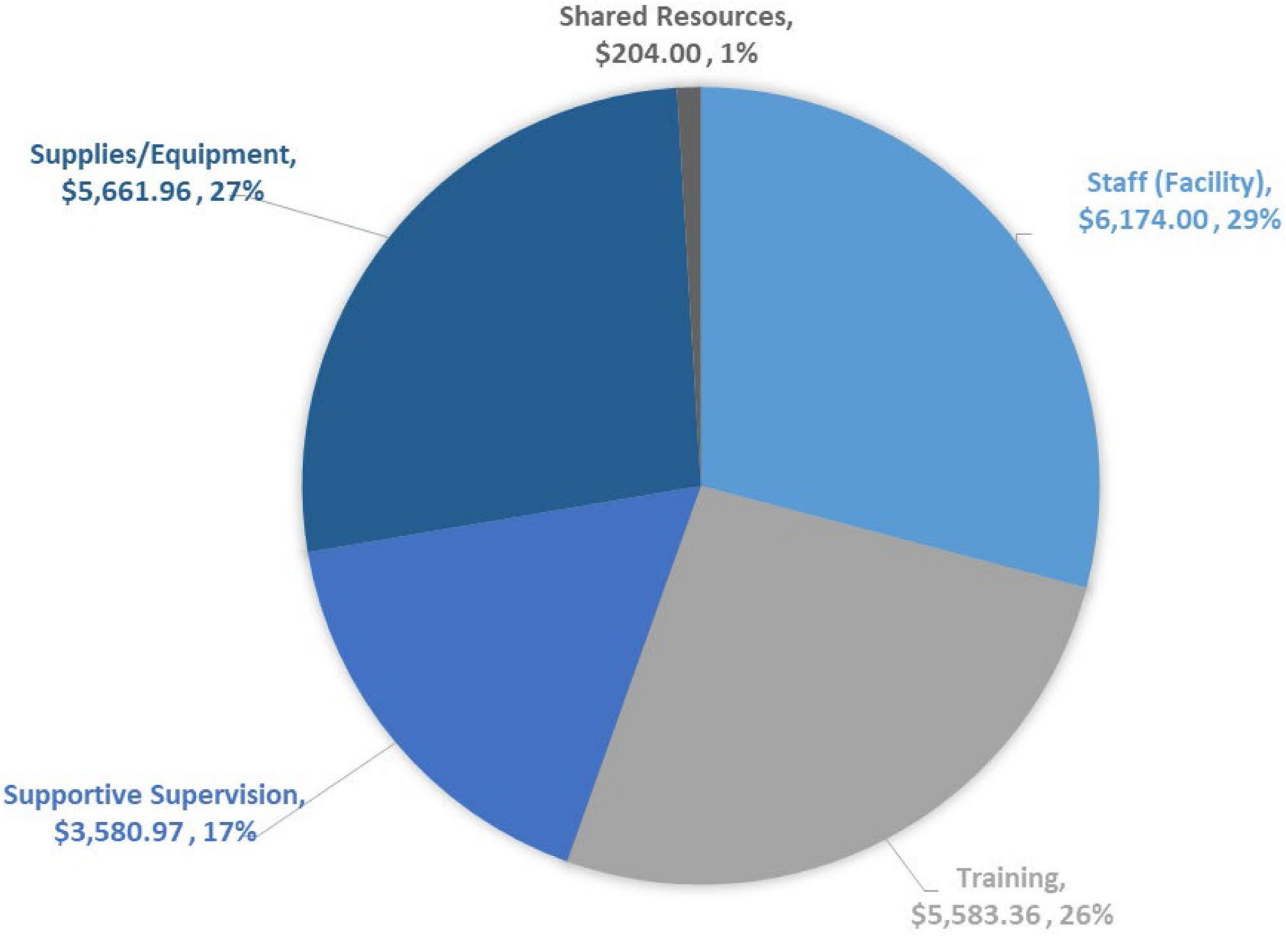
Project total cost by expenditure category in US Dollars. Fig. 3 is a pie chart that shows the percent contribution and total US dollar value of each cost category for the intervention

Costs were calculated by category and percent of total as follows: facility staff ($5,136.24, 25.3%), training ($5,583.36, 27.6%), supportive supervision ($3,652.97, 18.0%), supplies and equipment ($5,661.96, 28.0%), and shared resources ($204.00, 1.02%) (Fig. 3). Costs to support facility staff time were similar in all facilities ranging from $1201.80-1345.20. Training costs varied across sites, ranging from a total of $249.60 ($4.89 per participant) at Facility 2 to $2202.72 ($46.87 per participant) at Facility 4, taking into account travel, lodging and compensation costs such as per diem. Supportive supervision costs were primarily travel and lodging costs in order to provide technical assistance and implementation guidance by headquarters staff based in the US Embassy, and varied from a low of $72.00 at Facility 2 to a high of $1,644.27 at Facility 1. For supplies and equipment such as PPE, boots, sanitizer, thermometers and spray bottles, we calculated the cost of actual units used rather than the cost for total units procured and distributed. These costs ranged from $646.74 ($7.70 per participant) at Facility 3 to $2912.76 ($40.45 per participant) at Facility 1. Shared resources costs for this analysis were low overall and included only the percent of internet access charges used for the project. The bulk of shared resource costs, such as bills for water, sewer and electricity, were donated in-kind by NMOD-HIP and could not be disaggregated, in line with the intent of IPC integration into existing services. Costs associated with US Embassy staff time for activities such as protocol development, monitoring, procurement and distribution, financial activities, and data management were donated in-kind as part of established PEPFAR program costs for technical assistance related to HIV service delivery.

## Discussion

After implementing an IPC intervention in select Nigerian military tertiary facilities nearly one year into the COVID-19 pandemic, there was no improvement in overall IPC readiness as evidenced by a slight decline of 1.5% since the baseline score of 81.2%. However, IPC readiness varied considerably across modified WHO core IPC program components, certain individual questions, assessment time points, and facilities. Readiness was consistently high over time in the component of ‘IPC guidelines, policies, and SOPs’. ‘Infrastructure’ and ‘monitoring and remediation’ needed improvement at baseline and improved somewhat by follow-up. Although baseline scores were high across ‘IPC program structure, funding and leadership engagement’, ‘training, knowledge and practice’, and ‘biosafety and waste management’, follow-up scores declined considerably. ‘PPE materials availability and adequacy’ improved, while ‘triage and screening’ remained the same at follow-up with small improvements.

### Interpretation of findings

At baseline, all participating facilities reported having an IPC program with one or more dedicated IPC focal persons, but none reported this position was full time, which is consistent with recent survey results from other low-income countries and that could lead to other facility-wide deficits [20]. Surprisingly, by follow-up, Facility 3 reported no IPC program nor designated focal person despite having an IPC committee, and facilities 3 and 4 reported that facility leadership was no longer routinely meeting with the IPC committee or focal person. Although we did not conduct further assessment beyond the follow-up questionnaire, we speculate that this may have been tied to standard military service rotations or personnel transfer out to facilities in the military health system where COVID-19 burden and IPC needs were greater by follow-up assessment, or perhaps due to attrition, reprioritization of resources for other urgent clinical needs, or COVID-19 fatigue a year into the pandemic. Funding for IPC was not formally budgeted for at the health facility level in 2021, which has been reported to be problematic in other low-income healthcare settings [18, 19]. The annual cost for implementing an IPC intervention or program was low in the context of PLHIV services delivery, suggesting an opportunity to improve structure and funding of IPC programs in Nigerian military tertiary facilities for patient care and safety, HCW safety, and pandemic preparedness.

Participating facilities were successful in ensuring the availability of IPC policies, guidelines and SOPs across a range of applicable topics at follow-up. This success was attributed to the incident command and control structure instituted by NMOD-HIP at the outset of the pandemic, as well as the PEPFAR CQI culture that promotes the accessibility of policies, guidelines and SOPs [20, 21]. These findings were a stark contrast to WHO findings from a survey of other low-income countries where missing SOPs were common [18].

We found that the infrastructure component scored lowest at follow-up, which is consistent with other Nigerian reports and the global literature on IPC-related infrastructure in low-income countries [18, 22]. Ventilation in patient waiting areas was one reason for this. Challenges with proper ventilation in waiting areas and lack of an air filtration system are features that are common in facilities in the low or middle-income country setting [23]. This was reflected in our interventional reliance on administrative controls such as masking and creating additional waiting area space outdoors under tents. These efforts were in addition to preexisting routine practices in line with national COVID-19 policy, including use of standing fans, opening of windows for cross ventilation, and appropriate triage of respiratory illness patients. In the laboratory setting, use of air extractor fans was a requirement.

Conversely, functional hand hygiene stations were available at key facility points for all facilities. A Nigerian survey of water sanitation and hygiene in 2019 found that only 65% of urban facilities had improved water access on premises, and 70% had hand hygiene facilities at points of care and water and soap at toilets [24]. Furthermore, 51% of urban facilities segregated, treated and disposed of waste safely, whereas all facilities we assessed had access to functional waste treatment and documented waste management procedures [24]. That said, Facility 3 reported insufficient waste bins for segregation of waste at follow-up, which serves as a reminder that ongoing assessment, quality improvement and sufficient funding are required to ensure continued adherence to IPC best practices and to replace supplies and materials over time.

While we asked a different set of questions, our tertiary facility findings are similar to a primary health care setting intervention implemented across 22 African nations including Nigeria, where IPC gaps were commonly cited and measures of screening and triage declined [25]. Our facilities reported employing triage procedures, signage, screening of HCWs, visitors and patients at entrance for COVID-19 symptoms. Yet when it came to using tools like checklists and/or maintaining records of triage and screening, facilities fell short. Notably, documentation of HCW screening was not consistently maintained at follow-up, and facility 2 reported no longer screening HCWs daily with or without documentation. In high- and middle-income countries, digital syndromic surveillance tools that rely on self-administered screening checklists were adopted early in healthcare settings and other businesses for COVID-19 screening, risk profiling and triaging. In African countries, this form of surveillance was not widespread, and was primarily performed within smaller subsets of populations as research, with study teams administering screening questions and capturing self-reported information. Outside of these settings save for a handful of countries, self-screening has largely lacked documentation and relied on voluntary self-reporting of symptoms, exposure and testing to national systems. The lack of documentation and/or response may be due to stigma, concern for loss of income, and perceived level of importance of self-reporting symptoms, among other reasons [26, 27].

Despite some variation at project outset, all sites had some basic IPC training which may be attributed to military’s experience with EVD and Lassa fever outbreaks [9, 10]. These efforts included in-service training of HCWs in IPC generally. The NMOD-HIP also trained over 400 HCWs on COVID-19-specific IPC measures early in the pandemic [20]. Further opportunity exists to build upon this foundational knowledge and direct IPC focal persons and others into established IPC certification pathways such as through the Infection Control African Network, or enhance knowledge through open source resources such as the OpenWHO core IPC competency trainings [18, 28]. In the military context, meeting the frequency of new training needs due to high turnover driven by military rotations and duty tours remains an ongoing operational challenge. The decline reported in training at Facility 2 in relation to biosafety and waste management could reflect this.

Although continuous medical masking has been shown to significantly decrease HCWs’ risk for respiratory infection in healthcare facilities in areas with community transmission of COVID-19, sub-optimal PPE use may sustain risk of transmission [29–31]. Practical, in-person training that includes PPE donning, use and doffing is essential and was included in our intervention. Interactive IPC training for HCWs and nursing home staff with emphasis on medical devices and hand hygiene can enhance knowledge and improve patient outcomes [32]. In a 2020 international survey of nearly 3000 HCWs from different cadres, the majority had never received formal PPE training, which was strongly associated with low confidence in PPE use [33].

In comparison to a study of Nigerian civilian primary care facilities where PPE availability decreased over time, the availability of PPE in our tertiary facilities generally increased overall or was maintained [26]. Contributing factors could include the variability in PPE availability throughout the pandemic, the difference in timing of interventions, or the possibility of improved PPE access at the tertiary facility level versus primary care, and the deliberate inclusion of PPE distribution in our intervention [34–36]. However, facilities reported inconsistent internal distribution to staff working outside of COVID-19 treatment areas, a problem that has been reported in other regions globally and which aligns with US CDC and Nigerian CDC guidance in 2020 about PPE resourcing according to three levels (conventional, contingency, crisis) of operational status [37–39]. We speculate that internal rationing may have occurred, which would have been out of step with facility-level policy as well as PPE stockpiles reported to be available during intervention. Regardless of why, this underscores the importance of PPE commodity security for pandemic preparedness and response.

Kimani et al. 2022 found that only 38% of 777 health facilities surveyed in Kenya during the first year of the COVID-19 pandemic reported routinely monitoring HCW IPC practices [40]. In general, we found that monitoring and remediation activities such as audits of adherence to IPC best practices and SOPs were occurring at baseline and continued through to follow-up. However, conducting audits using routinized tools was an area for improvement, and one facility surprisingly did not meet any of the minimum criteria for this component at either baseline or follow-up. Despite PEPFAR’s strong culture of CQI, other IPC activities may have taken precedence over monitoring and remediation at this facility during a rapidly evolving pandemic, reflecting local and cultural priorities and determinants [41].

### Public health implications

Our intervention helped inform PEPFAR’s recently revised strategic direction. *Reimagining PEPFAR’s Strategic Direction: Fulfilling America’s Promise to End the HIV/AIDS Pandemic by 2030* outlines five pillars including one on public health systems and security [42]. Pillar foci include strengthening regional and national public health institutions, protecting the health workforce, and leveraging PEPFAR assets to improve health systems resilience and responsiveness while sustaining HIV services and impact. In line with this revised strategy, IPC has been established as one of the PEPFAR core standards for all country programs receiving funding, and integrated into PEPFAR’s facility assessment CQI tool (Site Improvement through Monitoring) [21].

Efforts to improve IPC readiness programmatically and ensure consistent uptake can be facilitated through integration into ongoing CQI activities. Since 2021, the IPC needs assessment questionnaire with stoplight scoring has been integrated into routine facility assessments across Nigerian military facilities. The results and tools also helped inform the inclusion of IPC formally into an existing PEPFAR facility assessment tool across PEPFAR-supported national HIV programs for quality improvement. Additional budgetary support from both PEPFAR and NMOD-HIP was allocated in the second half of 2021 to support assessment and intervention across an expanded number of facilities. This approach to embedding IPC components as routine practice with continuous monitoring and remediation could help continue to keep IPC program costs low, ensure dedicated resources in program budgets, and help maintain program fidelity over time, even as outbreaks and pandemics wane.

### Strengths and limitations

Our work had several strengths, including the modification of an established IPC framework that afforded our intervention consistency and reliability, the use of a stoplight color scheme consistent with existing CQI activities to facilitate staff understanding, and deliberate inclusion of HCW cadres from various disciplines to promote a ‘community’-level response and safety. Additionally, NMOD-HIP had strong prior experience in management of EVD and Lassa outbreaks as well as research and program evaluation. The intervention was embedded within the existing financial and technical assistance for HIV service delivery, and the NMOD-HIP had a command and control structure that provided an ideal environment to quickly test an intervention that might be rolled out to other Nigerian facilities and PEPFAR-supported national HIV programs.

Globally, changes in policies and guidelines were continual and often occurred in response to the availability of funding, material resources and emerging scientific evidence during the COVID-19 pandemic. Notably, our intervention was conducted nearly a year into the COVID-19 pandemic in 2021 in the context of an existing program focused on HIV service delivery and the first phase of COVID-19 vaccine availability in Nigeria; thus, results reflect IPC readiness mid-pandemic. By that time, the Nigerian Federal Ministry of Health and Social Welfare had already made available national IPC policies specific to COVID-19 which the NMOD pushed out to its facilities. The Nigerian military had also taken other major operational and guidance measures to protect HCWs and PLHIV, including establishing COVID-19 isolation and treatment centers, procuring PPE, and providing prior COVID-19 IPC training. The mature partnership between PEPFAR, MHRP, and NMOD-HIP had had time to draw upon and reallocate existing lines of funding to shore up IPC measures, including prioritizing this proof of concept intervention for testing. Given this context and timing it is not surprising that there were no reports of, e.g. PPE stock-outs, which had plagued hospitals around the world in the first year of the pandemic. In fact, the baseline results of this work were used to inform requests to PEPFAR and NMOD for additional IPC funding that was made available in late 2021, which also permitted expansion of efforts to other facilities. Thus, findings from this work must be carefully considered in relation to this mid-pandemic context, as they neither reflect the ‘worst case’ scenario of the early pandemic, nor the learning and subsequent expanded funding. However, the timing may have importantly permitted the identification and remediation of challenges such as supply tracking, distribution and accessibility across departments within facilities, which otherwise might not have been apparent at pandemic outset when procurement challenges were rampant.

Our work also had several important limitations. Collection of COVID-19 HCW testing results and isolation and quarantine outcomes were outside the scope of this project given the urgency of associated need and our program evaluation approach which was not intended to cover collection of protected health information. Instead, we focused on comparing baseline and follow-up IPC readiness, which we felt was a reasonable approach for an initial project, and which afforded lessons that could be applied to other settings and when scaling.

Our follow-up results showed declines in IPC readiness across three of eight components, and no change in a fourth, although this masked some improvements and declines in readiness at facility level. It is possible that awareness of IPC and related practices was improved in the context of the IPC intervention, empowering respondents to be more critical of their facility IPC program at follow-up. As the assessment tool was designed to be used by an outside party working in conjunction with the IPC focal person for the facility, we cannot exclude the possibility of respondent bias in our results, nor that the source of the information did not adequately capture the changes made. COVID-19 fatigue mid-pandemic may have also played a role.

While the assessment content was based on long-standing, globally-established IPC literature, tools and thematic areas of relevance to COVID-19 IPC, in hindsight some questions were less useful to guiding intervention development or were not actionable if gaps were identified due to the funding and scope limitations of the project. For example, a question in the ‘infrastructure’ section asked about environmental ventilation including natural, mechanical and UV irradiation to purify air to reduce COVID-19 and other respiratory infection transmission. Mechanical ventilation and UV irradiation methods were not relevant facility-wide. Rather, they were only available to COVID-19 treatment centers that were not included in the IPC intervention. Further, we were unable to remediate the gap fully where inadequate natural and mechanical ventilation were observed and documented in participating departments, which would have required additional project funding.

## Conclusion

Variability in sustaining intervention elements across the facilities over time highlights the importance of building and maintaining resilience in the face of pandemic-related stress, and the need for additional continuous quality improvement to identify interventions and strategies that successfully do so. Employing a quality improvement approach to IPC necessarily includes regular monitoring, re-assessment and re-training at set intervals. Results can be used to encourage solutions-oriented dialogue between staff and leadership, determine priority needs and implement appropriate action plans to improve IPC practices that protect staff. Successes, as well as employing context-relevant tools to determine needs to meet minimum requirements for IPC, can be used to advocate for dedicated resources towards IPC to elevate practices, improve outbreak preparedness and minimize disruptions in health services with an eye toward long-term health system resilience. Further research is needed to examine challenges with internal distribution of PPE throughout a facility during a pandemic, beyond isolation and treatment areas.

### Electronic supplementary material

Below is the link to the electronic supplementary material.

Supplementary Material 1

Supplementary Material 2

## Data Availability

Data are available from the authors upon reasonable request and with permission of the Nigerian Ministry of Defence-Health Implementation Programme.

## Declarations

### Disclaimer

The views expressed are those of the authors and should not be construed to represent the positions of the U.S. Army, the Department of Defense, the Uniformed Services University of the Health Sciences, the Henry M Jackson Foundation for the Advancement of Military Medicine, the Department of State or the U.S. Centers for Disease Control and Prevention. The investigators have adhered to the policies for protection of human subjects as prescribed in Army Regulation 70 − 25.

## References

[1] Infection prevention and control Geneva, Switzerland: World Health Organization. ; 2023 [Available from: https://www.who.int/health-topics/infection-prevention-and-control#tab=tab_1.

[2] Minimum requirements for infection prevention and control programmes. Geneva: Switzerland: World Health Organization; 2019.

[3] Weekly Bulletin on Outbreaks and Other Emergencies (2020). Week 25 15–21 June 2020.

[4] Elimian KO, Ochu CL, Ilori E, Oladejo J, Igumbor E, Steinhardt L (2020). Descriptive epidemiology of coronavirus Disease 2019 in Nigeria, 27 February-6 June 2020. Epidemiol Infect.

[5] Joy Okwor T, Gatua J, Umeokonkwo CD, Abah S, Ike IF, Ogunniyi A (2022). An assessment of Infection prevention and control preparedness of healthcare facilities in Nigeria in the early phase of the COVID-19 pandemic (February-May 2020). J Infect Prev.

[6] Holtzman CW, Godfrey C, Ismail L, Raizes E, Ake JA, Tefera F (2022). PEPFAR’s role in protecting and leveraging HIV services in the COVID-19 response in Africa. Curr HIV/AIDS Rep.

[7] Golin R, Godfrey C, Firth J, Lee L, Minior T, Phelps BR (2020). PEPFAR’s response to the convergence of the HIV and COVID-19 pandemics in Sub-saharan Africa. J Int AIDS Soc.

[8] Bailey LE, Siberry GK, Agaba P, Douglas M, Clinkscales JR, Godfrey C (2021). The impact of COVID-19 on multi-month dispensing (MMD) policies for antiretroviral therapy (ART) and MMD uptake in 21 PEPFAR-supported countries: a multi-country analysis. J Int AIDS Soc.

[9] Nwafor CD, Ilori E, Olayinka A, Ochu C, Olorundare R, Edeh E (2021). The One Health approach to incident management of the 2019 Lassa Fever outbreak response in Nigeria. One Health.

[10] Kwaja CMA, Olivieri DJ, Boland S, Henwood PC, Card B, Polatty DP, et al. Civilian perception of the role of the military in Nigeria’s 2014 Ebola outbreak and health-related responses in the North East region. BMJ Military Health. 2021. bmjmilitary-2020-001696. 10.1136/bmjmilitary-2020-001696 33547194

[11] CDC Facilities COVID-19 Screening. Atlanta, GA: US Centers for Disease Control and Prevention; 2020.

[12] Infection prevention (2018). And control assessment framework at the facility level.

[13] COVID-19 HOSPITAL PREPAREDNESS GUIDELINE. The Netherlands: SafeCare; 2020.

[14] Infection Prevention and Control Assessment Tool for Nursing Homes Preparing for COVID-19. Atlanta, GA: US Centers for Disease Control and Prevention; [Available from: https://www.cdc.gov/hai/prevent/infection-control-assessment-tools/nursing-homes.html.

[15] Rapid WASH, and IPC COVID-19 Health Facilities Assessment: Global WASH Cluster. ; 2020 [Available from: https://www.washcluster.net/covid-19/rapid-wash-and-ipc-covid-19-health-facilities-assessment.

[16] Site Improvement through Monitoring System (SIMS) (2020). 4.1 Site Level Master CEE Library.

[17] COVID-19 Situation Report (2021). Weekly Epidemiological Report 13. Abuja.

[18] Tomczyk S, Twyman A, de Kraker MEA, Coutinho Rehse AP, Tartari E, Toledo JP (2022). The first WHO global survey on Infection prevention and control in health-care facilities. Lancet Infect Dis.

[19] Qureshi M, Chughtai A, Seale H. Supporting the delivery of Infection Prevention and Control Training to Healthcare Workers: insights from the Sector. Healthc (Basel). 2022;10(5). 10.3390/healthcare10050936 PMC9141703 35628072

[20] Ayemoba O, Adekanye U, Iroezindu M, Onoh I, Lawal I, Suleiman A (2022). The Nigerian Military Public Health Response to COVID-19: a 14-Month Appraisal. Health Secur.

[21] Site Improvement through Monitoring System (SIMS) (2022). 4.2 Site Level Master CEE Library.

[22] Olayinka Stephen Ilesanmi OOA, Ayobami A, Bakare N, Adedosu A, Adeagbo A, Odutayo FO, Ayun, Ayomide E. Bello. Infection prevention and control (IPC) at a Lassa Fever treatment center before and after the implementation of an intensive IPC program. J Ideas Health. 2020;3(3).

[23] Ogunsola FT, Mehtar S (2020). Challenges regarding the control of environmental sources of contamination in healthcare settings in low-and middle-income countries - a narrative review. Antimicrob Resist Infect Control.

[24] Global progress. report on WASH in health care facilities: fundamentals first. World Health Organization; 2020.

[25] Patel LN, Kozikott S, Ilboudo R, Kamateeka M, Lamorde M, Subah M (2021). Safer primary healthcare facilities are needed to protect healthcare workers and maintain essential services: lessons learned from a multicountry COVID-19 emergency response initiative. BMJ Global Health.

[26] Ogboghodo EO, Osaigbovo II, Obarisiagbon OO, Okwara BU, Obaseki DE, Omo-Ikirodah OT (2021). Facility-based surveillance activities for COVID-19 Infection and outcomes among Healthcare Workers in a Nigerian Tertiary Hospital. Am J Trop Med Hyg.

[27] Bielicki JA, Duval X, Gobat N, Goossens H, Koopmans M, Tacconelli E (2020). Monitoring approaches for health-care workers during the COVID-19 pandemic. Lancet Infect Dis.

[28] OpenWHO course catalogues Geneva, Switzerland: World Health Organization. ; 2022 [Available from: https://openwho.org/pages/catalogues#infection-prevention-and-control-channel-2.

[29] Seidelman JL, Lewis SS, Advani SD, Akinboyo IC, Epling C, Case M (2020). Universal masking is an effective strategy to flatten the severe acute respiratory coronavirus virus 2 (SARS-CoV-2) healthcare worker epidemiologic curve. Infect Control Hosp Epidemiol.

[30] Suen LKP, Guo YP, Tong DWK, Leung PHM, Lung D, Ng MSP (2018). Self-contamination during doffing of personal protective equipment by healthcare workers to prevent Ebola transmission. Antimicrob Resist Infect Control.

[31] Evans S, Agnew E, Vynnycky E, Stimson J, Bhattacharya A, Rooney C (2021). The impact of testing and Infection prevention and control strategies on within-hospital transmission dynamics of COVID-19 in English hospitals. Philosophical Trans Royal Soc B: Biol Sci.

[32] Koo E, McNamara S, Lansing B, Olmsted RN, Rye RA, Fitzgerald T (2016). Making Infection prevention education interactive can enhance knowledge and improve outcomes: results from the targeted Infection Prevention (TIP) study. Am J Infect Control.

[33] Tabah A, Ramanan M, Laupland KB, Buetti N, Cortegiani A, Mellinghoff J (2020). Personal protective equipment and intensive care unit healthcare worker safety in the COVID-19 era (PPE-SAFE): an international survey. J Crit Care.

[34] Martin CA, Pan D, Nazareth J, Aujayeb A, Bryant L, Carr S (2022). Access to personal protective equipment in healthcare workers during the COVID-19 pandemic in the United Kingdom: results from a nationwide cohort study (UK-REACH). BMC Health Serv Res.

[35] Burki T (2020). Global shortage of personal protective equipment. Lancet Infect Dis.

[36] Oladele DA, Idigbe IE, Musa AZ, Gbaja-Biamila T, Bamidele T, Ohihoin AG (2021). Self-reported use of and access to personal protective equipment among healthcare workers during the COVID-19 outbreak in Nigeria. Heliyon.

[37] Martin-Delgado J, Viteri E, Mula A, Serpa P, Pacheco G, Prada D (2020). Availability of personal protective equipment and diagnostic and treatment facilities for healthcare workers involved in COVID-19 care: a cross-sectional study in Brazil, Colombia, and Ecuador. PLoS ONE.

[38] Cohen J, Rodgers YVM (2020). Contributing factors to personal protective equipment shortages during the COVID-19 pandemic. Prev Med.

[39] Livingston E, Desai A, Berkwits M (2020). Sourcing Personal Protective Equipment during the COVID-19 pandemic. JAMA.

[40] Kimani D, Ndegwa L, Njeru M, Wesangula E, Mboya F, Macharia C (2022). Adopting World Health Organization Multimodal Infection Prevention and Control Strategies to Respond to COVID-19, Kenya. Emerg Infect Dis.

[41] Birgand G, Johansson A, Szilagyi E, Lucet JC (2015). Overcoming the obstacles of implementing Infection prevention and control guidelines. Clin Microbiol Infect.

[42] Reimagining PEPFAR, s Strategic Direction (2022). Fulfilling America’s Promise to End the HIV/AIDS pandemic by 2030.

