# Supplementary material for: Protecting healthcare workers and patients during the COVID-19 pandemic: a comparison of baseline and follow-up infection prevention and control needs in Nigerian military healthcare facilities delivering HIV services
Source: BMC Health Serv Res. 2023 Nov 14;23:1254. doi: 10.1186/s12913-023-10289-x (PMC10647028; doi:10.1186/s12913-023-10289-x)
Supplement: Supplementary file 1 — Supplementary Material 1 [file 12913_2023_10289_MOESM1_ESM.pdf]

Additional file 1: Color-coded and scored results of infection prevention and control components overall and by facility at baseline and follow-up.

Component description	Facility 1		Facility 2		Facility 3		Facility 4		All Facilities (n=4)	
	Baseline	Follow-Up	Baseline	Follow-Up	Baseline	Follow-Up	Baseline	Follow-Up	Baseline	Follow-Up
Leadership	78.6%	85.7%	92.9%	71.4%	92.9%	28.6%	92.9%	57.1%	89.3%	60.7%
IPC policies, guidelines and Standard Operating Procedures	93.3%	100.0%	100.0%	100.0%	100.0%	86.7%	93.3%	100.0%	96.7%	96.7%
Infrastructure	66.7%	66.7%	50.0%	66.7%	58.3%	50.0%	50.0%	50.0%	56.3%	58.3%
Triage and screening	60.0%	60.0%	86.7%	66.7%	80.0%	80.0%	66.7%	86.7%	73.3%	73.3%
Training, knowledge and practice	100.0%	100.0%	80.0%	40.0%	100.0%	60.0%	60.0%	80.0%	85.0%	70.0%
Personal protective equipment materials availability & adequacy	77.8%	100.0%	69.4%	100.0%	72.2%	55.6%	100.0%	100.0%	79.9%	88.9%
Biosafety and waste management	100.0%	100.0%	100.0%	75.0%	100.0%	75.0%	100.0%	100.0%	100.0%	87.5%
Monitoring and remediation	0.0%	0.0%	80.0%	80.0%	100.0%	100.0%	70.0%	100.0%	62.5%	70.0%
Overall Facility Performance	74.0%	81.3%	82.7%	81.3%	85.3%	68.0%	82.7%	88.0%	81.2%	79.7%

Key				
	0-25%	26-50%	51-75%	76-100%
